# The Fos-Related Antigen 1–JUNB/Activator Protein 1 Transcription Complex, a Downstream Target of Signal Transducer and Activator of Transcription 3, Induces T Helper 17 Differentiation and Promotes Experimental Autoimmune Arthritis

**DOI:** 10.3389/fimmu.2017.01793

**Published:** 2017-12-18

**Authors:** Young-Mee Moon, Seon-Yeong Lee, Seung-Ki Kwok, Seung Hoon Lee, Deokhoon Kim, Woo Kyung Kim, Yang-Mi Her, Hea-Jin Son, Eun-Kyung Kim, Jun-Geol Ryu, Hyeon-Beom Seo, Jeong-Eun Kwon, Sue-Yun Hwang, Jeehee Youn, Rho H. Seong, Dae-Myung Jue, Sung-Hwan Park, Ho-Youn Kim, Sung-Min Ahn, Mi-La Cho

**Affiliations:** ^1^Laboratory of Immune Network, The Rheumatism Research Center, College of Medicine, Catholic Research Institute of Medical Science, The Catholic University of Korea, Seoul, South Korea; ^2^Center for Rheumatic Disease, Division of Rheumatology, Department of Internal Medicine, Seoul St. Mary’s Hospital, College of Medicine, The Catholic University of Korea, Seoul, South Korea; ^3^Asan Institute for Life Sciences, University of Ulsan College of Medicine, Asan Medical Center, Seoul, South Korea; ^4^Department of Pathology, Asan Medical Center, University of Ulsan College of Medicine, Seoul, South Korea; ^5^Department of Developmental Biology, Washington University School of Medicine, St. Louis, MO, United States; ^6^Department of Chemical Engineering, Hankyong National University, Anseong, South Korea; ^7^Department of Biomedical Sciences, College of Medicine, Hanyang University, Seoul, South Korea; ^8^Department of Biological Sciences, Institute of Molecular Biology and Genetics, Research Center for Functional Cellulomics, Seoul National University, Seoul, South Korea; ^9^Department of Biochemistry, College of Medicine, The Catholic University of Korea, Seoul, South Korea; ^10^Department of Hemato-oncology, Bioinformatics, Cancer Research, Systems Biology, Gachon University, Seongnam, South Korea

**Keywords:** Fos-related antigen 1-JUNB, signal transducer and activator of transcription 3, T helper 17, autoimmune arthritis, inflammation

## Abstract

Dysfunction of T helper 17 (Th17) cells leads to chronic inflammatory disorders. Signal transducer and activator of transcription 3 (STAT3) orchestrates the expression of proinflammatory cytokines and pathogenic cell differentiation from interleukin (IL)-17-producing Th17 cells. However, the pathways mediated by STAT3 signaling are not fully understood. Here, we observed that Fos-related antigen 1 (FRA1) and JUNB are directly involved in STAT3 binding to sites in the promoters of *Fosl1* and *Junb*. Promoter binding increased expression of IL-17 and the development of Th17 cells. Overexpression of *Fra1* and *Junb* in mice resulted in susceptibility to collagen-induced arthritis and an increase in Th17 cell numbers and inflammatory cytokine production. In patients with rheumatoid arthritis, FRA1 and JUNB were colocalized with STAT3 in the inflamed synovium. These observations suggest that FRA1 and JUNB are associated closely with STAT3 activation, and that this activation leads to Th17 cell differentiation in autoimmune diseases and inflammation.

## Introduction

T helper 17 (Th17) cells are a pathogenic subset of T helper lymphocytes that play a key role in inflammatory disorders leading to a severe chronic immune inflammatory response. Th17 cells release several proinflammatory cytokines including interleukin (IL)-17A, IL-21, and IL-22 (1). IL-17A is an inflammatory cytokine produced predominantly by Th17 cells with strong effects on stromal cells. IL-17A induces inflammatory cytokine production and leukocyte recruitment, which act to link the innate and adaptive branches of immunity (2). Both IL-17A and Th17 cells are highly involved in the pathogenesis of several autoimmune diseases, including rheumatoid arthritis (RA) (3, 4), while playing a significant role in autoimmune disorders mediated by excessive inflammation.

Signal transducer and activator of transcription 3 (STAT3) is an important transcription factor with DNA-binding properties whose activity is paramount to the inflammatory response. It has been suggested that STAT3 activation upregulates IL-17 production *via* Th17 cell proliferation (5, 6). Several transcription factors including STAT3 regulate Th17 cell differentiation (7–12); however, STAT3 also plays a key role in the immune inflammatory response. There is a general consensus that STAT3 is essential for Th17 cell differentiation (13). Moreover, STAT3 modulates the production of several cytokines including IL-17A and activates downstream transcription factors, such as RAR-related orphan receptor gamma isoform 2 (RORγt), which is responsible for the Th17 phenotype (14, 15).

The activator protein 1 (AP-1) family is a group of structurally and functionally related JUN (c-JUN, JUNB, and JUND) and FOS [c-FOS, FOSB, Fos-related antigen 1 (FRA1), and FRA2] transcription factors. AP-1 heterodimers are involved in a variety of biological processes including cell proliferation, differentiation, apoptosis, and inflammation (16, 17). It has been suggested that AP-1 proteins are involved in several pathological conditions (18–21), while JUN and FOS proteins are also associated with the immune inflammatory response. Modulation of c-FOS and c-JUN expression is critical for inhibition of IL-17 production (22) and the maintenance of suppressive regulatory T-cell function (23). Additionally, production of FRA1, a member of the FOS protein family, is increased by B cell stimulation (24). Furthermore, JUNB modulates the proliferation of B cells (25). This evidence suggests that FRA1 and JUNB may be involved in regulating the inflammatory immune response.

We hypothesized that FRA1 and JUNB modulate the Th17 cell-mediated inflammatory response. The aim of this study was to elucidate whether FRA1 and JUNB regulate autoimmune arthritis *via* Th17 cell differentiation and factors downstream of STAT3. We used *in vitro* models, *in vivo* animal models, and clinical specimens from patients with RA to investigate the biological importance of this pathway.

## Materials and Methods

### Mice

Collagen-induced arthritis (CIA) was induced in 6–8-week-old male DBA/1J, BALB/c, and C57BL/6 mice (Orient, Korea). To generate *Fra1/Junb* Tg mice, a pcDNA3.1+HA (Invitrogen, CA, USA) vector containing the FRA1 and JUNB proteins coupled to a linker peptide (3 × GGGGS) was constructed. The *Fra1/Junb* fragment was synthesized by GenScript Corporation (NJ, USA), with codon optimization for expression in mammalian cells. *Fra1/Junb* Tg mice were bred from the C57BL/6 line and maintained in facilities at Macrogen Laboratories (Seoul, Korea). All mice were maintained under specific-pathogen-free conditions at the Institute of Medical Science, The Catholic University of Korea. The presence of the transgene in the founders was confirmed by PCR of genomic DNA extracted from the tail samples. Genotyping was performed by PCR analysis of genomic DNA obtained from mice at 3 weeks of age. All experimental procedures were examined and approved by the Animal Research Ethics Committee at the Catholic University of Korea.

### Accession Codes

Raw RNA-seq data have been deposited in the NCBI Sequence Read Archive (SRR6320798 and SRR6320799).

A detailed description of all other experimental procedures and the statistical analysis is provided in the Section “Supplementary Materials and Methods” in Data Sheet S1 in Supplementary Material.

## Results

### STAT3 Target Genes Are Differentially Expressed in Mouse Th17 Cells

**Figure 1:**
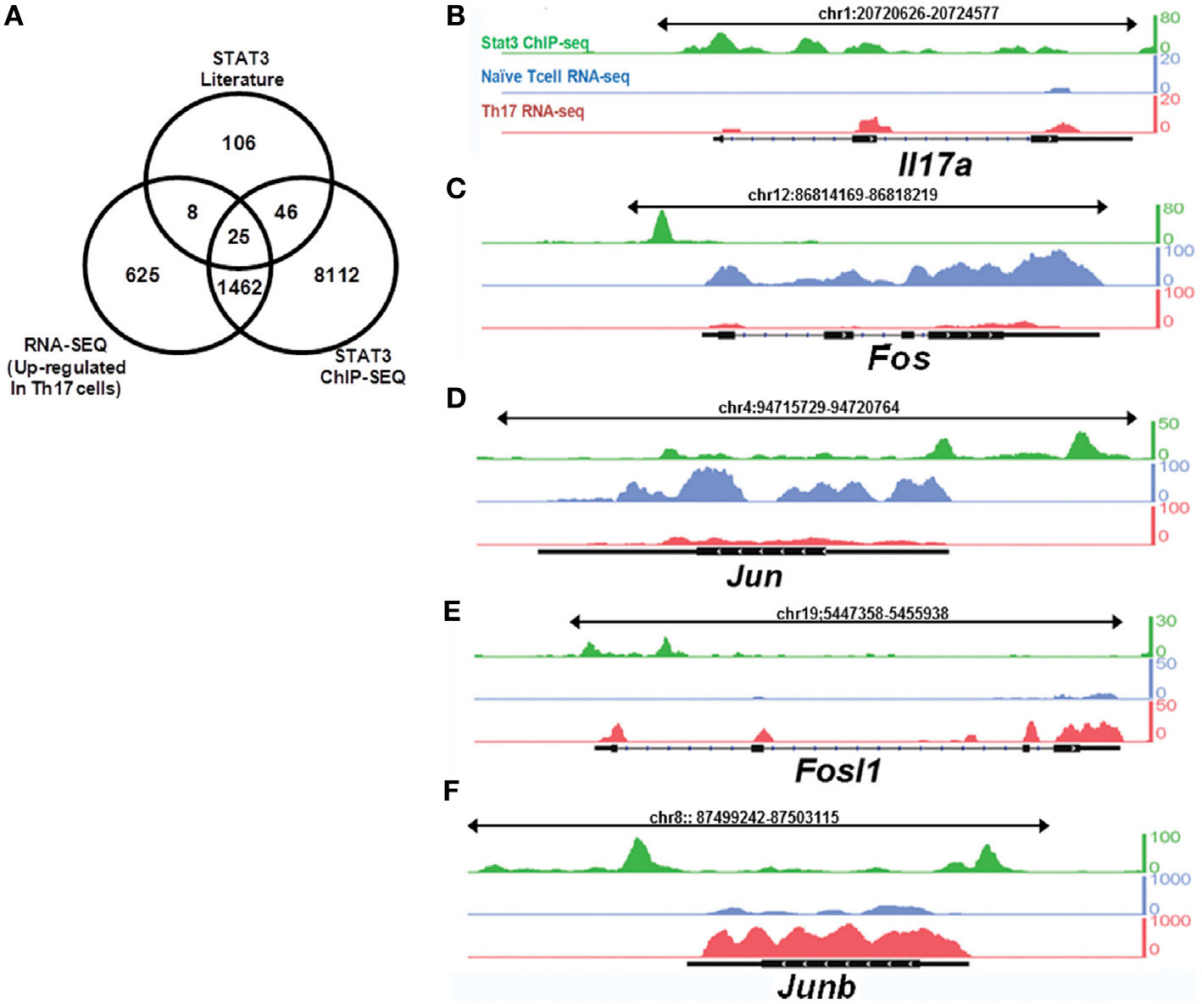
Signal transducer and activator of transcription 3 (STAT3) target genes are differentially expressed in naïve and T helper 17 (Th17) cells. Naïve T cells (CD4^+^CD62L^+^) were isolated from 6-week-old mice (*n* = 3), stimulated under Th17-polarizing conditions for 3 days, and then sorted for CD4^+^CD62L^−^ expression. RNA was sequenced using the Illumina GA IIx. STAT3 chromatin immunoprecipitation sequencing (ChIP-Seq) data for CD4^+^ T cells were downloaded from the NCBI Sequence Read Archive (SRP002451). **(A)** A comparison of differentially expressed genes identified by RNA-Seq, STAT3-binding target genes identified by ChIP-Seq, and the downstream targets of STAT3. The expression of **(B)** *IL17a* and **(C–F)** activator protein 1 transcription factor target genes [**(C)** *Fos*, **(D)** *Jun*, **(E)** *Fosl1*, and **(F)** *Junb*] was compared between naïve T cells and Th17 cells, with reference to STAT3-binding target genes (obtained from STAT3 ChIP-Seq data in Th17 cells). The peak profiles are color-coded as follows: naïve T cells (RNA-Seq: blue), Th17 cells (RNA-Seq: red), and STAT3-binding genes (ChIP-Seq: green).

Potential STAT3-binding sites were identified using publicly available chromatin immunoprecipitation sequencing (ChIP-Seq) data cross-referenced with differentially expressed genes in Th17 cells (14). We sequenced mRNA obtained from Th17 cells and naïve T cells and compared the results with potential STAT3-binding targets identified by ChIP-Seq to identify STAT3-regulated genes involved in Th17 cell differentiation. The literature was systematically reviewed for downstream STAT3 targets in humans and mice in multiple biological contexts. By combining these three datasets, we searched for genes with STAT3-binding sites that are upregulated during Th17 cell differentiation (Figure 1A; Table S1 in Supplementary Material). RNA-Seq was performed to identify genes differentially expressed between Th17 cells and naïve T cells. This integrated approach suggested that *Fosl1* (the gene locus of *Fra1*) and *Junb* are Th17 cell differentiation factors downstream of STAT3. As shown in Figures 1B–F and Table S2 in Supplementary Material, *Fos* and *Jun* were the predominant AP-1 subtypes expressed in naïve (CD4^+^CD62L^+^) T cells, whereas *Fosl1* and *Junb* were the predominant subtypes in Th17 cells. Other AP-1 subtypes were downregulated, suggesting that *Fosl1* and *Junb* AP-1 subtypes play an important role in Th17 cell differentiation.

### *Fra1* and *Junb* Are Highly Expressed in Th17 Cells, and Their Expression Is Regulated Directly by STAT3 in Mice

**Figure 2:**
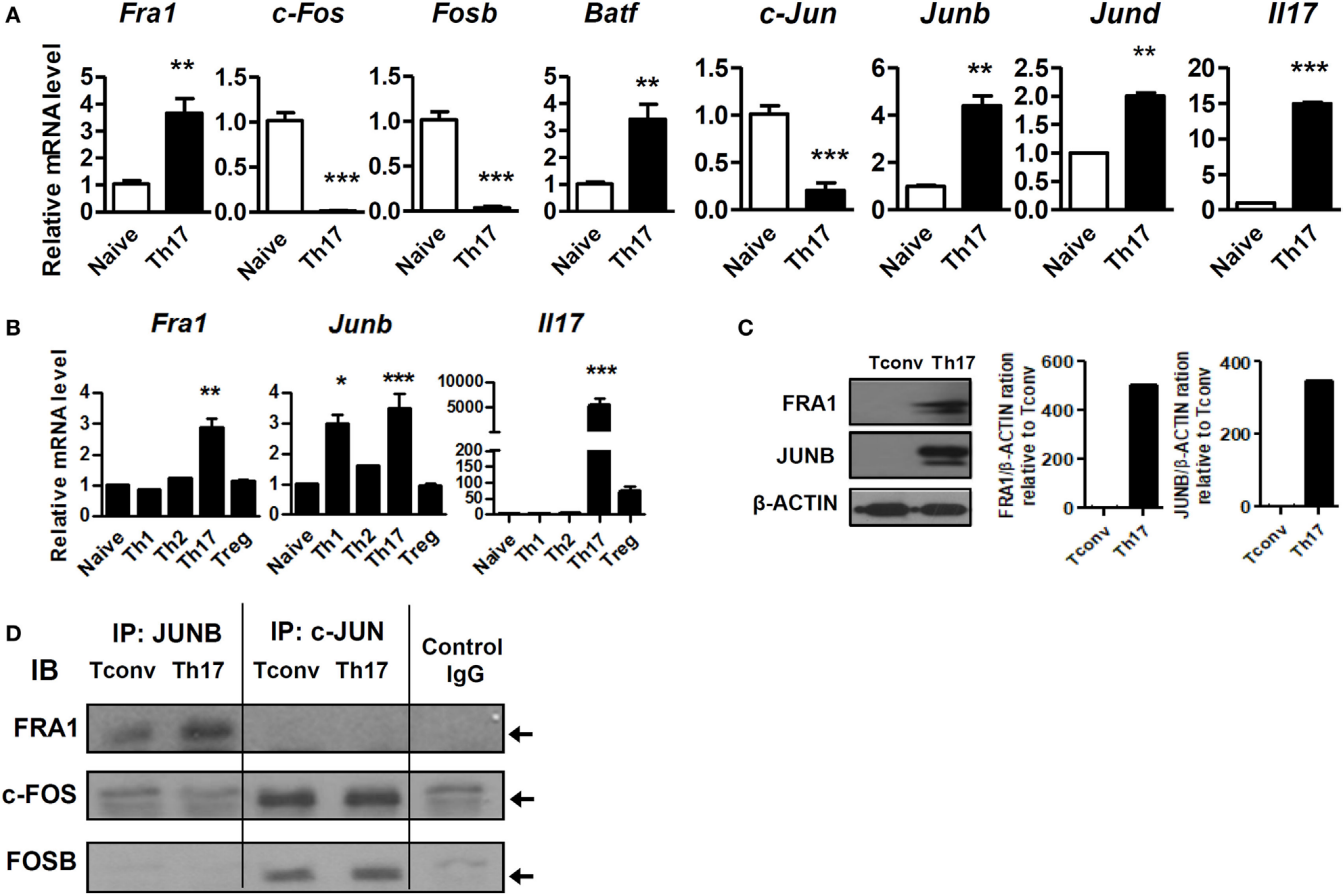
Both Fos-related antigen 1 (FRA1) and JUNB are highly expressed in T helper 17 (Th17) cells. **(A,B)** Naïve T cells (CD4^+^CD62L^+^) were isolated from 6-week-old C57BL/6 mice (*n* = 3) and cultured for 3 days under the indicated promoters. Relative mRNA levels were measured by qRT-PCR. **(C)** Protein extracts were prepared from CD4^+^ T cells (Tconv) and CD4^+^ T cells cultured for 3 days under Th17-polarizing conditions (Th17). FRA1 and JUNB protein levels were evaluated by immunoblotting. **(D)** CD4^+^ T cells cultured under Th17-polarizing conditions were lysed, and the cell lysates were subjected to co-immunoprecipitation with anti-JUNB and anti-c-JUN antibodies and immunoblotting with anti-FRA1, anti-c-FOS, and anti-FOSB antibodies. Data are representative of three **(A–C)** or two **(D)** independent experiments, each performed in triplicate **(A,B)**. The data were analyzed using an unpaired *t-*test or one-way ANOVA with Bonferroni’s *post hoc* test. Error bars show the SEM. *p < 0.05, **p < 0.005, and ***p < 0.0005.

Quantitative real-time reverse transcription PCR (qRT-PCR) and immunoblot analyzes were used to confirm *Fra1* and *Junb* expression. Among the various AP-1 subtypes, only *Fra1* and *Junb* mRNA levels were significantly increased in Th17 cells compared with naive T cells (Figures 2A,B). These findings supported the RNA-Seq results. Immunoblot analysis also showed high levels of FRA1 and JUNB expression in Th17 cells (Figure 2C). Co-immunoprecipitation studies were performed using antibodies against Jun family proteins (i.e., JUNB and c-JUN), followed by immunoblotting using antibodies against FOS family proteins (i.e., FRA1, c-FOS, and FOSB) to determine whether FRA1 and JUNB form a heterodimeric AP-1 complex. It was found that FRA1 forms a complex with JUNB, but not with c-JUN (Figure 2D).

**Figure 3:**
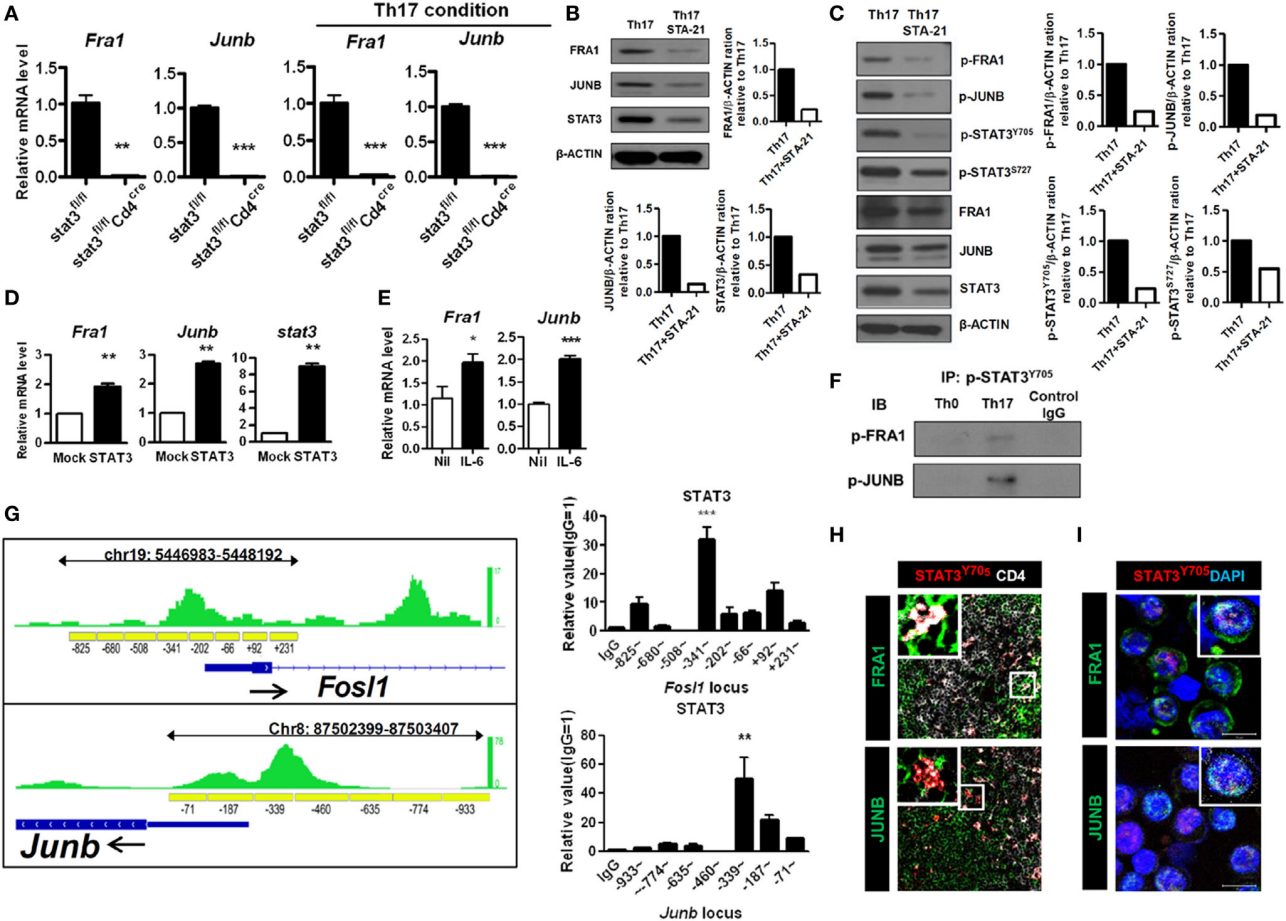
Signal transducer and activator of transcription 3 (STAT3)-mediated activation of Fos-related antigen 1 (FRA1) and JUNB in T helper 17 (Th17) cells. **(A)** CD4^+^ T cells from *Stat3*^fl/fl^ and *Stat3*^fl/fl^Cd4-Cre were isolated (left panel) or cultured under Th17-polarizing conditions for 3 days (right panel). **(B,C)** CD4^+^ T cells were pretreated with STA-21 for 1 day, cultured under Th17-polarizing conditions for a further 3 days, and then treated with interleukin (IL)-6 for 2 h **(C)** before harvesting and lysing. **(D)** LBRM cells were transfected with a STAT3 expression vector, and the cells were cultured for 3 weeks in the presence of neomycin (10 µg/ml). **(E)** CD4^+^ T cells were cultured in the presence of IL-6 for 16 h. **(F)** CD4^+^ T cells were cultured under Th0- or Th17-polarizing conditions for 3 days and the lysed. The cell lysates were subjected to immunoprecipitation with an anti-phospho-STAT3^Y705^ antibody and immunoblotting with anti-phospho-FRA1 or anti-phospho-JUNB antibodies. **(G)** The STAT3-binding region (green) detected by STAT3 chromatin immunoprecipitation (ChIP) sequencing in Th17 cells is shown along with the *Fosl1* and *Junb* loci, including the primer sites (yellow boxes) (left panel). The negative control cyclophilin showed no enrichment (data not shown). CD4^+^ T cells were cultured under Th17-polarizing conditions for 3 days. ChIP-qPCR analysis was performed using an anti-STAT3 antibody and primers specific for *Fosl1* and *Junb* (right panel). **(H,I)** Splenic tissue was collected from collagen-induced arthritis mice. CD4+ splenic T cells and Th17 cells from arthritic mice were stained for CD4 (white), FRA1, JUNB (green), and phospho-STAT3^Y705^ **(H)** or DAPI **(I)** (blue). Data are representative of three **(B–I)** and two **(A)** independent experiments, each performed in triplicate **(A,D,E,G)**. The data were analyzed using an unpaired *t-*test; error bars show the SEM. *p < 0.05, **p < 0.005, and ***p < 0.0005.

Next, we investigated the functional association between the FRA1–JUNB complex and STAT3. The STAT3 signaling pathway was inhibited using genetically engineered STAT3-deficient CD4^+^ T cells and STA-21, which blocks the DNA-binding activity of STAT3 (26). As shown in Figure 3A, CD4^+^ T cells harboring *Stat3*^fl/fl^*Cd4*-Cre had markedly decreased levels of *Fra1* and *Junb* mRNA under both neutral and Th17-polarizing conditions. Treatment with STA-21 profoundly decreased the expression of FRA1, JUNB, and STAT3 (Figure 3B). Moreover, STA-21 decreased the levels of STAT3 phosphorylation under Th17-polarizing conditions (Figure 3C). STAT3 overexpression increased the relative levels of *Fra1* and *Junb* mRNA (Figure 3D), while *in vitro* treatment with IL-6 upregulated *Fra1* and *Junb* mRNA (Figure 3E).

Co-immunoprecipitation studies were performed using anti-FRA1 and anti-JUNB antibodies followed by immunoblotting with antibodies against STAT3 to determine whether FRA1 and JUNB physically interact with STAT3. Both FRA1 and JUNB were found to form a complex with STAT3 (Figure 3F). We performed ChIP and qPCR to examine whether STAT3 binds directly to the promoter regions of *Fosl1* and *Junb*. The ChIP-qPCR results were compared with publicly available ChIP-Seq data from Th17 cells. We found that STAT3 binds to the predicted recognition sites upstream of *Fosl1* and *Junb*, suggesting that it regulates their expression by binding directly to their promoters (Figure 3G). Using confocal microscopy, we found that FRA1 and JUNB were colocalized with phosphorylated STAT3 in CD4^+^ splenic T cells and Th17 cells from arthritic mice (Figures 3H,I). These findings suggest that STAT3 signaling is critical for the expression and activation of FRA1 and JUNB during Th17 cell differentiation.

### Expression of IL-17 Is Directly Regulated by the FRA1–JUNB Complex

**Figure 4:**
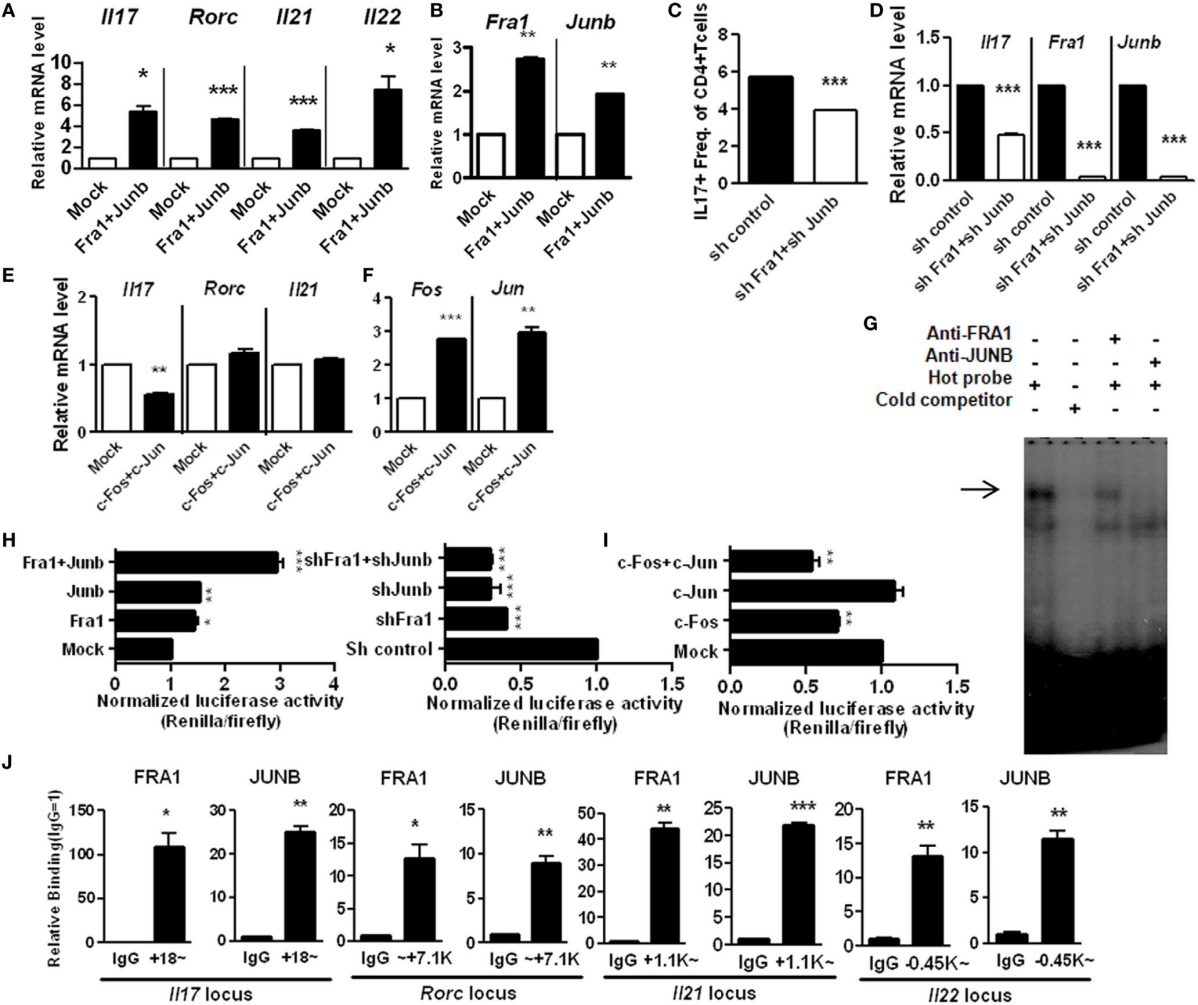
Expression of interleukin (IL)-17 is directly mediated by the Fos-related antigen 1 (FRA1)–JUNB complex in T helper 17 (Th17) cells. LBRM cells were transfected with vectors and cultured for 3 weeks with neomycin (10 µg/ml) **(A,B,E,F)**. **(A,B)** The relative mRNA levels of each gene were measured by qRT-PCR. **(C,D)** CD4^+^ T cells were infected with lentiviruses harboring constructs designed to knockdown *Fra1* and *Junb*. Cells were then cultured under Th17-polarizing conditions for 3 days. CD4^+^IL-17^+^ T cells were identified by flow cytometry, and relative mRNA levels were measured by qRT-PCR. **(E,F)** The relative mRNA level of each gene was measured by qRT-PCR. **(G)** Nuclear extracts from Th17 cells were analyzed by electrophoretic mobility shift assay incorporating a radiolabeled probe derived from the activator protein 1-binding site of the *IL17a* locus and antibodies specific for FRA1 or JUNB. **(H,I)** EL4 cells were transiently transfected with expression vectors and *IL17a* promoter constructs with a CNS2 enhancer region. Luciferase activity was measured using a dual-luciferase reporter assay system. **(J)** CD4^+^ T cells were cultured under Th17-polarizing conditions. Chromatin immunoprecipitation-qPCR was performed using anti-FRA1 or anti-JUNB antibodies with primers specific for each gene locus. Data are representative of at least three independent experiments, performed in triplicate. Data were analyzed using an unpaired *t-*test; error bars show the SEM. *p < 0.05, **p < 0.005, and ***p < 0.0005.

The AP-1 family plays a versatile role in T-cell development (27). Levels of IL-17 were measured in *Fra1*- and *Junb*-overexpressing and silenced cells. The relative levels of *IL17* mRNA were significantly increased in cells overexpressing *Fra1* and *Junb*. The relative mRNA levels of other genes associated with Th17 cells, such as *IL21, IL22*, and *Rorc* (the RORγt gene locus), were also increased (Figures 4A,B). CD4^+^IL-17^+^ cells and *IL17* mRNA levels were significantly decreased when *Fra1* and *Junb* were silenced under Th17-polarizing conditions (Figures 4C,D). By contrast, when *c-Fos* and *c-Jun*, the dominant AP-1 subtypes in naïve T cells, were overexpressed, *IL17* mRNA levels were decreased significantly (Figures 4E,F). These findings support the specificity of the role of the FRA1–JUNB complex in Th17 cell differentiation.

We next investigated whether the FRA1–JUNB complex directly regulates IL-17 expression by binding to the *IL17a* promoter. The interaction between the FRA1–JUNB complex and the AP-1-binding motif within the *IL17a* promoter region was examined by electrophoretic mobility shift assay. The anti-FRA1 and anti-JUNB antibodies caused a super shift of the labeled DNA probe, indicating that the AP-1-binding motif interacts specifically with FRA1 and JUNB (Figure 4G). This finding was confirmed by an analysis of the *IL17a* promoter using a luciferase reporter system. Overexpression of both FRA1 and JUNB increased luciferase activity in EL4 cells. Simultaneous overexpression of FRA1 and JUNB caused luciferase activity to increase twofold (Figure 4H). Silencing of *Fra1* and *Junb* caused a greater than twofold reduction in luciferase activity. Simultaneous overexpression of c-FOS and c-JUN caused a significant decrease in the luciferase activity induced by the *IL17a* promoter (Figure 4I). ChIP-qPCR analyses showed that FRA1 and JUNB bind directly to the *IL17a* gene loci and to gene loci encoding other Th17 cell differentiation activators, such as *IL21, IL22*, and *Rorc* (Figure 4J). These findings indicate that the FRA1–JUNB complex binds to the *IL17a* promoter region, and that this binding activates *IL17* gene transcription.

### FRA1 and JUNB Are Critical for the Pathogenesis of CIA

**Figure 5:**
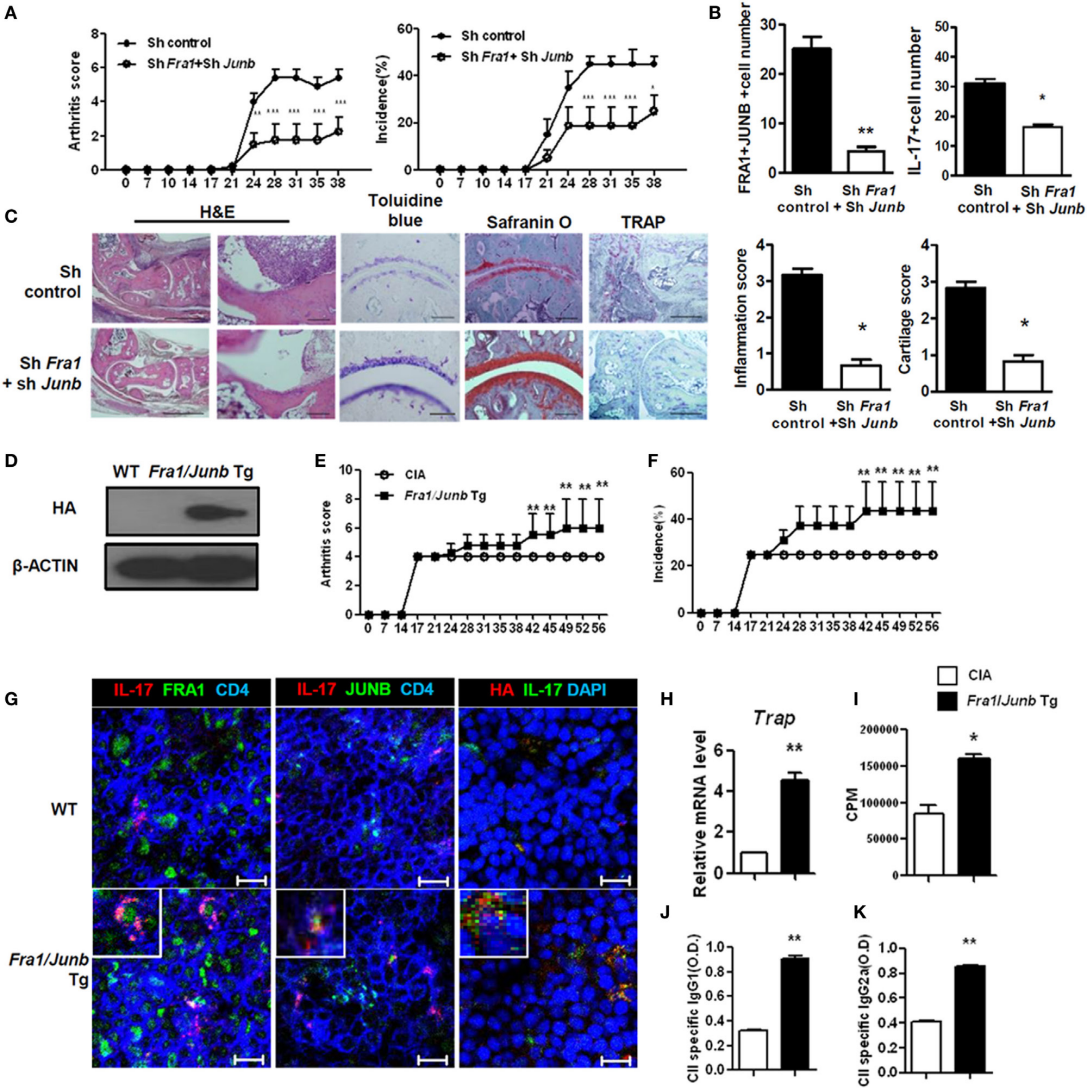
Fos-related antigen 1 (FRA1) and JUNB are involved in the pathogenesis of collagen-induced arthritis, T helper 17 cell differentiation, and rheumatoid inflammation in mice. Arthritis was induced in mice, and shRNA control or shRNA*Fra1*/shRNA*Junb* vectors were injected on days 7 and 14 (*n* = 5–7). **(A)** Arthritis severity was measured using the incidence and mean arthritis score. **(B)** Splenic tissue from each mouse was stained. Positively stained cells were counted visually by four individuals from high-magnification images projected onto a screen, and the mean values are presented as a histogram. **(C)** On day 30 after immunization, tissues were sampled from the ankle joints and stained with toluidine blue, Safranin O (scale bar = 100 µm), H&E [scale bar = 500 µm (left); 100 µm (right)], or tartrate-resistant acid phosphatase (TRAP) (scale bar = 200 µm). The inflammation and cartilage scores are shown in bar graphs (right panel). **(D)** Splenocytes were examined for the HA-tagged FRA1/JUNB transgene by immunoblotting. **(E,F)** Disease severity was recorded using the incidence and mean arthritis score. **(G)** Splenic tissue from each mouse was stained for FRA1, JUNB, and interleukin-17 in CD4^+^ T cells and examined by laser confocal microscopy. **(H)** The relative levels of TRAP mRNA in cells from the ankle joints were measured by qRT-PCR. **(I)** T-cell proliferation was analyzed using a mixed-lymphocyte reaction. **(J,K)** The levels of IgG1 and IgG2a antibodies specific to type II collagen were measured in serum. Data are representative of more than two independent experiments. Data were analyzed using an unpaired *t-*test or two-way ANOVA with Bonferroni’s *post hoc* test; error bars show the SEM. *p < 0.05, **p < 0.005, and ***p < 0.0005.

T helper 17 cells and IL-17 contribute significantly to the development of RA (28, 29). To examine the *in vivo* role of the FRA1–JUNB complex in rheumatoid inflammation, *Fra1* and *Junb* were silenced in CIA mice by injecting *Fra1* and *Junb* short hairpin RNA vectors. This markedly reduced the incidence of arthritis and decreased the arthritis score (Figure 5A). The numbers of FRA1^+^, JUNB^+^, and IL-17^+^ cells were significantly lower within the joint tissues (Figure 5B). Histological examination showed that the ankle joints of *Fra1/Junb*-silenced mice had less inflammation and cartilage damage (Figure 5C). Inhibition of *Fra1/Junb* also reduced the expression of Th17 cytokines such as IL-6, IL-17, IL-1β, and tumor necrosis factor-α in joint tissue. Other mediators of joint destruction such as vascular endothelial growth factor and receptor activator of nuclear factor κB ligand (RANKL) were also downregulated (Figure S1A in Supplementary Material). In CIA mice, disease severity was correlated with the level of type II collagen (CII)-specific IgG antibodies (30). Silencing of *Fra1* and *Junb* reduced T-cell proliferation and CII-specific antibody production (Figures S1B,C in Supplementary Material). It also substantially reduced the population of IL-17-producing CD4^+^ T cells, considered to be Th17 cells, in the spleen and draining lymph nodes (Figure S1D in Supplementary Material).

CD4^+^ T cells in *Fra1*/*Junb* Tg mice had different proportions of Th1 and Th17 cells (Figure S2 in Supplementary Material). CIA was induced in *Fra1/Junb* transgenic mice to investigate the *in vivo* effects of *Fra1* and *Junb* overexpression. Splenocytes were examined for HA-tagged FRA1/JUNB transgene by immunoblotting (Figure 5D). The incidence of arthritis and the arthritis score were significantly increased (Figures 5E,F). There was a marked increase in the levels of IL-17, FRA1, and JUNB in the spleen (Figure 5G). The relative mRNA level of the osteoclastogenic marker tartrate-resistant acid phosphatase was significantly higher (Figure 5H), while CII-specific T-cell proliferation and IgG production were also increased (Figures 5I–K). Overexpression of FRA1/JUNB profoundly increased CIA joint pathology (Figure S3A in Supplementary Material). Immunostaining showed that the expression of various proinflammatory cytokines and RANKL, a mediator of osteoclastogenesis, was significantly greater in the joint tissues of transgenic mice with CIA (Figure S3B in Supplementary Material). IL-17 mRNA levels and Th17 cell numbers were increased markedly (Figures S3C,D in Supplementary Material). These findings suggest that the FRA1–JUNB complex plays a major role in the development of CIA, likely through the induction of Th17 cell differentiation.

### Expression and Potential Functions of FRA1 and JUNB in RA Patients

**Figure 6:**
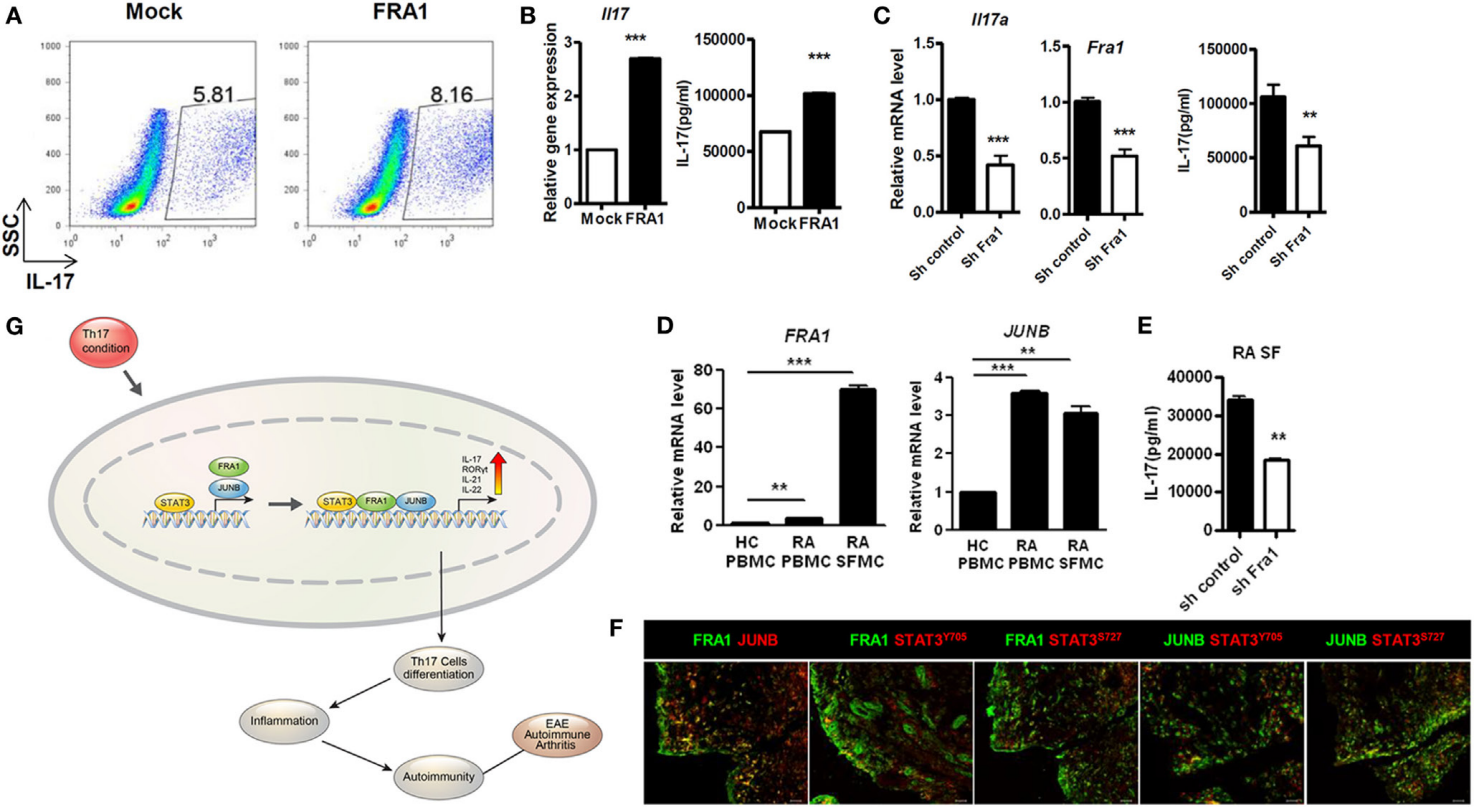
Expression and potential function of Fos-related antigen 1 (FRA1) and JUNB in rheumatoid arthritis (RA) patients. **(A,B)** Flow cytometry, qRT-PCR, and ELISA. CD4^+^ T cells were isolated from healthy peripheral blood mononuclear cells (PBMCs) and infected with retroviruses containing FRA1 on day 1. The infected cells were then cultured under T helper 17 (Th17)-polarizing conditions for 3 days and analyzed. **(C)** qRT-PCR and ELISA. CD4^+^ T cells from healthy controls were infected with lentiviruses harboring constructs designed to knockdown *Fra1*. Cells were then cultured under Th17-polarizing conditions for 3 days. **(D)** qRT-PCR analysis. Total RNA was extracted from PBMCs isolated from healthy controls and from PBMCs and synovial fluid mononuclear cells (SFMCs) from RA patients. **(E)** ELISA. CD4^+^ T cells in SFMCs from RA patients were infected with lentiviruses harboring constructs designed to knockdown *Fra1*. Cells were then cultured under Th17-polarizing conditions for 3 days. **(F)** Synovial tissue obtained from RA patients. Sections were immunostained. Data are representative of three **(A–C,F)** and two **(D,E)** independent experiments, each performed in triplicate. **(G)** A schematic illustration of the inflammatory signaling pathway mediated by FRA1/JUNB. **(B–E)** Data are representative of more than three independent experiments. Data were analyzed using an unpaired *t-*test; error bars show the SEM. *p < 0.05, **p < 0.005, and ***p < 0.0005.

We demonstrated *in vivo* roles of FRA1 and JUNB in mice with CIA, an animal disease model for RA. To confirm the roles of FRA1 and JUNB in patients with RA, FRA1 was overexpressed in CD4^+^ T cells isolated from healthy controls. FRA1 overexpression increased the proportion of IL-17-producing CD4^+^ T cells during Th17 polarization (Figure 6A). These cells produced significantly higher levels of *IL17a* mRNA and cytokines (Figure 6B). *Fra1* silencing decreased *IL17a* mRNA levels and IL-17 secretion (Figure 6C). There were significantly increased the transcription levels of *Fra1* and *Junb* among peripheral blood mononuclear cells (PBMCs) and synovial fluid mononuclear cells (SFMCs) from RA patients compared with PBMCs from healthy controls (Figure 6D). *Junb* was also upregulated in RA patients. When *Fra1* was silenced in CD4^+^ T cells isolated from SFMCs of RA patients, IL-17 secretion was decreased during Th17 polarization (Figure 6E). The expression of both FRA1 and JUNB was particularly high in the inflamed synovium of RA patients, a region enriched with inflammatory immune cells (Figure 6F). Of note, FRA1, JUNB, and phosphorylated STAT3 were colocalized in the inflamed synovium, which further supported our *in vitro* experimental results. A schematic illustration of the inflammatory signaling pathway induced by FRA1/JUNB is shown in Figure 6G. Th17 conditioning induces an inflammatory signal by promoting FRA1/JUNB. The inflammatory response is mediated by activation of STAT3, which regulates genes involved in Th17 cell differentiation. This cascade induces excessive inflammation and autoimmunity resulting in RA.

## Discussion

Although FRA1 and JUNB may have modulatory activity in the immune inflammatory response (18, 19), there is currently no evidence in the literature to support this notion in autoimmune disorders mediated by STAT3 and Th17 cells. In this study, we found evidence of previously unidentified functions of FRA1 and JUNB in Th17 cell differentiation and autoimmune disease. We investigated the modulatory function and underlying mechanisms of FRA1 and JUNB in a murine model of inflammatory arthritis. The predominant conclusion from this study was that FRA1 and JUNB exacerbate inflammation *via* the induction of Th17 cells. To our knowledge, this is the first study to implicate FRA1 and JUNB directly in STAT3 activation and regulation of Th17 cell proliferation. These observations suggest a previously unidentified function of these factors in autoimmune disease.

Activator protein 1 is comprised of dimers of FOS and JUN protein family members (31, 32). The diverse functions of AP-1 family members result from heterodimer formation between the FOS and JUN proteins (33). AP-1 is also involved in cell functions including proliferation, differentiation, and apoptosis. For example, the FRA2–JUND complex is involved in the terminal differentiation of granulosa cells into luteal cells (34) and T-cell activation (35). Additionally, AP-1 factors may play important roles in autoimmune disease and Th17 cell differentiation. It was suggested that basic leucine zipper transcription factor, ATF-like (BATF), an AP-1 protein, leads to increased Th17 cell differentiation, whereas BATF deficiency results in IL-17 downregulation (12). Loss of the AP-1 family member Fosl2 in T cells improved EAE severity *via* suppression of Th17 cell plasticity (36). We performed an integrative analysis using RNA-Seq and ChIP-Seq data to identify key factors downstream of STAT3 involved in Th17 cell differentiation. This integrative analysis suggested that upregulation of *Fra1* and *Junb* drives and promotes Th17 cell differentiation.

Signal transducer and activator of transcription 3-mediated IL-17 expression and Th17 cell differentiation involve AP-1 family proteins. Previous studies have shown a biological link between the STAT3 signaling pathway and the AP-1 family of transcription factors (12, 37, 38). In particular, STAT3-mediated expression of BATF, an AP-1 subfamily member, is associated with Th17 cell differentiation (12). In this study, RNA-Seq analysis showed that the expression of *cFos* and *cJun* was higher in naïve T cells. This suggests that AP-1 subtype switching from c-FOS–c-JUN to FRA1/JUNB is essential for Th17 cell differentiation. It can also be postulated that activation of c-FOS–c-JUN must be suppressed in parallel with activation of FRA1/JUNB by STAT3 during polarization toward the Th17 subtype. The present study identified an AP-1 complex comprising FRA1 and JUNB, which appears to function as a key factor downstream of STAT3 in Th17 cell differentiation. It appears that AP-1 subtype switching, particularly from c-FOS, FOSB, and c-JUN to FRA1 and JUNB, is required for STAT3-mediated Th17 cell differentiation.

The AP-1 family includes the c-JUN and c-FOS proteins, which play important roles in IL-17 expression and Th17 cell functions. It has been demonstrated that BATF binds to intergenic components in the *IL17a* locus and to *IL17* promoters, both of which are essential for Th17 differentiation (12). Additionally, the BATF–JUNB and BATF–JUND complexes have been shown to cooperate with IRF4 in Th17 cells to promote transcription of *IL17* (39). The c-JUN transcription factor that heterodimerizes with c-FOS to generate the AP-1 transcription factor complex is involved in suppression of IL-17 production in developing Th17 cells (16, 22). FRA2 is also a negative regulator of IL-17 (36). We observed that overexpression of FRA2 decreased the expression of *IL17a* in a promoter-fused luciferase reporter assay (data not shown). We also found that expression levels of *c-Fos* and *c-Jun* were significantly reduced, whereas *Batf* expression was increased in Th17 cells compared with naïve T cells. Overexpression of *c-Fos* and *c-Jun* significantly decreased *IL17* mRNA levels. The FRA1–JUNB complex directly activates expression of IL-17, a cytokine produced by Th17 cells. This complex plays a role in promoting the expression of other cytokines associated with Th17 cells, such as IL-21 and IL-22, by directly binding to their promoter regions. These results suggest that FRA1 and JUNB are involved in IL-17 production and Th17 cell differentiation.

Although FRA1/JUNB and c-FOS/c-JUN compete for the same AP-1-binding sites within the *IL17a* promoter, we found in this study that *IL17a* promoter activity was increased by FRA1 and JUNB overexpression but decreased by c-FOS and c-JUN overexpression. We observed an interaction between p-STAT3 and p-FRA1 or p-JUNB in Th17 cells; therefore, FRA1/JUNB and c-FOS/c-JUN may be involved in AP-1 subtype switching. Additionally, p-FRA1 binds to p-JUNB in Th17 cells. Further study is needed to confirm the AP-1 subtype switching factors involved and the mechanism of interaction between p-FRA1 and p-JUNB in Th17 cells.

This study has certain limitations. One is that the animal studies were conducted using FRA1/JUNB-1 overexpression and knockdown vectors. Adoptive transfer studies in conditional overexpression and knockout mice are required to confirm that FRA1/JUNB-1 regulates CD4^+^ T cells, leading to autoimmune arthritis. Nonetheless, this study is the first to demonstrate functions of FRA1/JUNB-1 in Th17 cell differentiation and autoimmune arthritis. Future studies using adoptive transfer models with CD4^+^ T cells differentially expressing FRA1/JUNB-1 are required to validate our data more precisely.

Uncontrolled Th17 cell activation is responsible for the onset of several autoimmune diseases. Th17 cells cause tissue injury *via* production of IL-17, as observed in CIA mouse models of disease. The present study showed that the FRA1–JUNB complex plays a central role in the development of RA, which is a Th17-mediated autoimmune disease. The FRA1–JUNB complex may be clinically relevant as it functions as a Th17 cell-specific regulator of proinflammatory cytokine production in CIA mouse models and hence RA. In the RA mouse model used in this study, FRA1/JUNB overexpression resulted in the development of severe inflammatory symptoms and augmented expression of IL-17 in mice. Notably, this has fueled interest in the genetic importance of FRA1/JUNB in human RA in the context of promoted STAT3 activity. FRA1 overexpression activated Th17 cell differentiation in PBMCs from healthy controls; however, FRA1 silencing downregulated IL-17 production in SFMCs from RA patients. This may indicate the potential of the FRA1–JUNB complex as a therapeutic target in human autoimmune disease. In conclusion, our results suggest a previously unidentified function of FRA1/JUNB in T-cell-mediated autoimmune diseases. FRA1/JUNB directly modulates STAT3 activation and Th17 cell differentiation. Our identification of the involvement of FRA1/JUNB in T-cell development provides a potential therapeutic target for the treatment of autoimmune diseases.

## Ethics Statement

The Animal Care Committee of The Catholic University of Korea approved the experimental protocol. All experimental procedures were evaluated and carried out in accordance with the protocols approved by the Animal Research Ethics Committee at the Catholic University of Korea (CMCU-2012-0156-01). All procedures performed followed the ethical guidelines on animal use. Approval by the ethics committee of Seoul St. Mary’s Hospital (Seoul, Republic of Korea) was obtained for all procedures. All human experimental procedures were approved by the Ethics Committee of Seoul St. Mary’s Hospital (Seoul, Republic of Korea, KC13TISE0032).

## Author Contributions

Y-MM, S-YL, S-KK, SL, S-MA, and M-LC designed the experiments and analyzed the data. Y-MM, S-YL, S-KK, and SL wrote the manuscript along with input from DK, WK, Y-MH, H-JS, E-KK, J-GR, H-BS, J-EK, S-YH, and JY. Y-MM and S-YL performed all *in vitro* assays with help from S-KK, SL, DK, WK, and H-BS. S-YL, H-JS, and J-GR performed the animal experiments. E-KK conducted all immunohistochemistry experiments. Y-MM, S-YL, S-KK, SL, RS, D-MJ, H-YK, S-HP, S-MA, and M-LC discussed and developed the study concept. All authors critically reviewed and approved the final form of the manuscript.

## Conflict of Interest Statement

The authors declare that the research was conducted in the absence of any commercial or financial relationships that could be construed as a potential conflict of interest.

## Supplementary Material

Figure S1 Knockdown of FRA1/JUNB Decreases Th17 Cell Differentiation and Attenuates Rheumatoid Inflammation in Mice.

Figure S2 T cell proportion of CD4^+^Tcells in Fra1/Junb Tg mice.

Figure S3 Overexpression of FRA1/JUNB Causes Th17 Cell Differentiation and Increases CIA Disease Severity.
