# Supplementary material for: The Fos-Related Antigen 1–JUNB/Activator Protein 1 Transcription Complex, a Downstream Target of Signal Transducer and Activator of Transcription 3, Induces T Helper 17 Differentiation and Promotes Experimental Autoimmune Arthritis
Source: Front Immunol. 2017 Dec 18;8:1793. doi: 10.3389/fimmu.2017.01793 (PMC5741610; doi:10.3389/fimmu.2017.01793)
Supplement: Supplementary file 1 [file Table_1.PDF]

AP-1 Subtype Switching to FRA1-JUNB, a Downstream Target of STAT3, Induces Th17 Cell Differentiation and Experimental Autoimmune Arthritis

Young-Mee Moon[1]*, Seon-Yeong Lee[1]*, Seung-Ki Kwok[2]*, Seung Hoon Lee[1]*, Deokhoon Kim[3], Woo Kyung Kim[3], Yang-Mi Her[1], Hea-Jin Son[1], Eun-Kyung Kim[1], Jun-Geol Ryu[1], Hyeonbeom Seo[1], Jeong-Eun Kwon[1], Sue-Yun Hwang[4], Jeehee Youn[5] , Rho H. Seong[6], Sung-Hwan Park[2], Ho-Youn Kim[2], Sung-Min Ahn[3†], Mi-La Cho[1†]

[1] Laboratory of Immune Network, The Rheumatism Research Center, College of Medicine, The Catholic University of Korea, Seoul, 137-701, South Korea

[2] Center for Rheumatic Disease, Division of Rheumatology, Department of Internal Medicine, Seoul St. Mary's Hospital, College of Medicine, The Catholic University of Korea, Seoul, 137-701, South Korea.

[3] Asan Institute for Life Sciences, Asan Medical Center, University of Ulsan College of medicine, Seoul, Korea..

[4] Department of chemical engineering, Hankyong National University, Anseong, 456-749, South Korea.

[5] Department of Biomedical Sciences, College of Medicine, Hanyang University, Seoul 133-791, Korea

[6] Department of Biological Sciences, Institute of Molecular Biology and Genetics, Research Center for Functional Cellulomics, Seoul National University, Seoul 151-742, South Korea

Authorship note:

***** These authors contributed equally to this work

[†] These authors contributed equally to this work

Correspondence

†Mi-La Cho, PhD.

Rheumatism Research Center, College of Medicine, The Catholic University of Korea, 505 Banpo-dong, Seocho-gu, Seoul 137-701, Korea (South)

Phone: 82-2-2258-7467 , Fax: 82-2-599-4287

E-mail: iammila@catholic.ac.kr.

† Sung-Min Ahn, M.D, PhD

Department of Genome Medicine and Science, College of Medicine, Gachon University, Seongnam, Republic of Korea

Phone: 82-2-3010-3221 Fax: 82-2-2258-7473

E-mail: smahn@gachon.ac.kr

Supplementary tables

Table S1. Genes up-regulated by STAT3 in Th17 cells. These gene listed were from the STAT3 literature, STAT3 ChIP-seq, and those up-regulated in Th17 RNA-seq (see Figure 1a). RNA-seq FPKM values were from Cufflinks and ChIP-seq fold enrichment values were from MACS.

Genes	RNA-SEQ			ChIP-SEQ
	Naïve FPKM	Th17 FPKM	Log2 (Fold change)	Fold enrichment
SOCS1	26.40	301.4	3.51	7.36
SOCS3	19.68	84.07	2.09	14.6
CCND3	101.28	230.88	1.19	7.76
MT1	0	3.56	NA	10.23
FASN	36.06	72.94	1.02	5.93
BCL2L1	77.23	197.24	1.35	11.55
IL21	0.73	45.06	5.95	6.07
JUNB	122.6	676.44	2.46	13.53
HIF1A	54.64	148.16	1.44	10.38
MAP2K3	27.6	85.06	1.62	15.47
LDLR	14.57	33.89	1.22	8.97
OSM	11.52	34.17	1.57	10.02
IL2RA	121.31	365.91	1.59	9.21
MUC1	0	3.19	NA	12.66
TNF	20.94	64.97	1.63	7.21
CEBPB	4.56	39.31	3.11	12.3
NFIL3	23.69	73.72	1.64	5.02
TIMP1	0	14.12	NA	15.4
BIRC5	37.89	98.95	1.38	11.76
VIM	110.92	175.70	0.66	6.7
STRA13	31.71	68.26	1.11	6.16
JAK3	37.73	97.45	1.37	25.42
FOSL1	1.53	12.55	3.04	8.93
MYD88	28.79	57.56	1.00	17.63
GADD45B	13.7	41.96	1.61	7.19
