# Supplementary material for: The Fos-Related Antigen 1–JUNB/Activator Protein 1 Transcription Complex, a Downstream Target of Signal Transducer and Activator of Transcription 3, Induces T Helper 17 Differentiation and Promotes Experimental Autoimmune Arthritis
Source: Front Immunol. 2017 Dec 18;8:1793. doi: 10.3389/fimmu.2017.01793 (PMC5741610; doi:10.3389/fimmu.2017.01793)
Supplement: Supplementary file 2 [file Table_2.PDF]

AP-1 Subtype Switching to FRA1-JUNB, a Downstream Target of STAT3, Induces Th17 Cell Differentiation and Experimental Autoimmune Arthritis

Young-Mee Moon[1]*, Seon-Yeong Lee[1]*, Seung-Ki Kwok[2]*, Seung Hoon Lee[1]*, Deokhoon Kim[3], Woo Kyung Kim[3], Yang-Mi Her[1], Hea-Jin Son[1], Eun-Kyung Kim[1], Jun-Geol Ryu[1], Hyeonbeom Seo[1], Jeong-Eun Kwon[1], Sue-Yun Hwang[4], Jeehee Youn[5] , Rho H. Seong[6], Sung-Hwan Park[2], Ho-Youn Kim[2], Sung-Min Ahn[3†], Mi-La Cho[1†]

[1] Laboratory of Immune Network, The Rheumatism Research Center, College of Medicine, The Catholic University of Korea, Seoul, 137-701, South Korea

[2] Center for Rheumatic Disease, Division of Rheumatology, Department of Internal Medicine, Seoul St. Mary's Hospital, College of Medicine, The Catholic University of Korea, Seoul, 137-701, South Korea.

[3] Asan Institute for Life Sciences, Asan Medical Center, University of Ulsan College of medicine, Seoul, Korea..

[4] Department of chemical engineering, Hankyong National University, Anseong, 456-749, South Korea.

[5] Department of Biomedical Sciences, College of Medicine, Hanyang University, Seoul 133-791, Korea

[6] Department of Biological Sciences, Institute of Molecular Biology and Genetics, Research Center for Functional Cellulomics, Seoul National University, Seoul 151-742, South Korea

Authorship note:

***** These authors contributed equally to this work

[†] These authors contributed equally to this work

Correspondence

†Mi-La Cho, PhD.

Rheumatism Research Center, College of Medicine, The Catholic University of Korea, 505 Banpo-dong, Seocho-gu, Seoul 137-701, Korea (South)

Phone: 82-2-2258-7467 , Fax: 82-2-599-4287

E-mail: iammila@catholic.ac.kr.

† Sung-Min Ahn, M.D, PhD

Department of Genome Medicine and Science, College of Medicine, Gachon University, Seongnam, Republic of Korea

Phone: 82-2-3010-3221 Fax: 82-2-2258-7473

E-mail: smahn@gachon.ac.kr

Supplementary tables

Table S2. Normalized expression of mRNA for the different AP-1 components, as assessed by RNA-seq. mRNAs for *Fra1* and *Junb* were up-regulated (orange) in Th17 cells. By contrast, most AP-1 containing *Fos* and *Jun* were down-regulated (green).

Genes	FPKM		Log2 FC	Significantly differential expression
	Naïve	Th17		
Fos	50.4073	8.59946	-2.551314706	Yes
Fosb	17.9181	0.888512	-4.333882594	Yes
Fosl1	1.52756	12.5539	3.038834669	Yes
Fosl2	7.80905	44.7291	2.517994775	Yes
Batf	65.3969	160.981	1.299596268	Yes
Atf1	23.0774	32.7524	0.505119934	No
Atf2	17.4291	12.4753	-0.482423565	No
Atf3	9.2181	33.6092	1.866314879	Yes
Atf4	209.046	462.911	1.146914407	Yes
Atf5	15.9971	24.03	0.587026256	Yes
Atf6	10.8498	7.95173	-0.448327773	No
Atf6b	28.8497	50.9526	0.820599449	No
Atf7	6.08665	1.29653	-2.23099282	No
Jun	32.4611	9.60697	-1.756558501	Yes
Junb	122.597	676.441	2.46404043	Yes
Jund	42.9703	278.62	2.696887067	Yes
