# Supplementary material for: The Fos-Related Antigen 1–JUNB/Activator Protein 1 Transcription Complex, a Downstream Target of Signal Transducer and Activator of Transcription 3, Induces T Helper 17 Differentiation and Promotes Experimental Autoimmune Arthritis
Source: Front Immunol. 2017 Dec 18;8:1793. doi: 10.3389/fimmu.2017.01793 (PMC5741610; doi:10.3389/fimmu.2017.01793)
Supplement: Supplementary file 3 [file Table_3.PDF]

AP-1 Subtype Switching to FRA1-JUNB, a Downstream Target of STAT3, Induces Th17 Cell Differentiation and Experimental Autoimmune Arthritis

Young-Mee Moon[1]*, Seon-Yeong Lee[1]*, Seung-Ki Kwok[2]*, Seung Hoon Lee[1]*, Deokhoon Kim[3], Woo Kyung Kim[3], Yang-Mi Her[1], Hea-Jin Son[1], Eun-Kyung Kim[1], Jun-Geol Ryu[1], Hyeonbeom Seo[1], Jeong-Eun Kwon[1], Sue-Yun Hwang[4], Jeehee Youn[5] , Rho H. Seong[6], Sung-Hwan Park[2], Ho-Youn Kim[2], Sung-Min Ahn[3†], Mi-La Cho[1†]

[1] Laboratory of Immune Network, The Rheumatism Research Center, College of Medicine, The Catholic University of Korea, Seoul, 137-701, South Korea

[2] Center for Rheumatic Disease, Division of Rheumatology, Department of Internal Medicine, Seoul St. Mary's Hospital, College of Medicine, The Catholic University of Korea, Seoul, 137-701, South Korea.

[3] Asan Institute for Life Sciences, Asan Medical Center, University of Ulsan College of medicine, Seoul, Korea..

[4] Department of chemical engineering, Hankyong National University, Anseong, 456-749, South Korea.

[5] Department of Biomedical Sciences, College of Medicine, Hanyang University, Seoul 133-791, Korea

[6] Department of Biological Sciences, Institute of Molecular Biology and Genetics, Research Center for Functional Cellulomics, Seoul National University, Seoul 151-742, South Korea

Authorship note:

***** These authors contributed equally to this work

[†] These authors contributed equally to this work

Correspondence

†Mi-La Cho, PhD.

Rheumatism Research Center, College of Medicine, The Catholic University of Korea, 505 Banpo-dong, Seocho-gu, Seoul 137-701, Korea (South)

Phone: 82-2-2258-7467 , Fax: 82-2-599-4287

E-mail: iammila@catholic.ac.kr.

† Sung-Min Ahn, M.D, PhD

Department of Genome Medicine and Science, College of Medicine, Gachon University, Seongnam, Republic of Korea

Phone: 82-2-3010-3221 Fax: 82-2-2258-7473

E-mail: smahn@gachon.ac.kr

Supplementary tables

Table S3. qPCR primers for ChIP-qPCR. The SABiosciences' proprietary database (DECODE) and Transcriptional Regulatory Element Database (TRED) were used to search for the proximal promoter regions or binding regions for transcription factors.

gene position	sense 5'~3'	antisense 5'~3'
Fosl1 +231	GCAAGCTCAGGCACAGACC	CGACCACCAGAGTCAGATGC
Fosl1 +92	GAGAGGCGCGAGTGAGTC	CGGTCTGTGCCTGAGCTT
Fosl1 -66	GACCGGTCCACAGAGGTTC	GCTGCGGTTCTGACTCACTC
Fosl1 -202	GCAAGGAAATTGCACCATGT	GACCCACCTCTCCAGATGAA
Fosl1 -341	CTCTCCCCGTTCTTCTTTCT	ACATGGTGCAATTTCCTTGC
Fosl1 -508	CAAACCACAAGCAAAGCAAA	TGTCTGAAAGCTGGGAAGTG
Fosl1 -680	CCAACCTGCACAACAAGAGA	TTTGCTTTGCTTGTGGTTTG
Fosl1 -825	AACCCTGGTTTGTCAATAACCTT	AGGTTGGCCTTGAACTCCTT
Junb -71	ACCACGGAGGGAGAGAAAAT	GAGCTGTCCTCGGCTGTG
*Jun*b-187	CCGCAGCTGACAAATTCAGT	ATTTTCTCTCCCTCCGTGGT
Junb -339	GCCGCTGTTTACAAGGACAC	GAATTTGTCAGCTGCGGAAG
Junb -460	TTCCGGGGATATCCTAAGGT	GTAAACAGCGGCCACGAG
Junb -635	ACTTTGTACCCGGGACCTCT	TATCCCCGGAAAAGTCTCGT
Junb -774	ATTGACCTCCCCAAAATTCC	AGAGGTCCCGGGTACAAAGT
Junb -933	CTCGGAAGGAAGCTATGTCG	GGAATTTTGGGGAGGTCAAT
Il17a +18	ACCTCACACGAGGCACAAGT	CCCAACAGCTGGAATAGAGC
Il21 +1092	CGCACAGCTAGGCCTTTAAT	TCATGTGCAGAGCTGGAGAG
Il22 -463	TGGCTTATTTCAAAGCACAGAA	TGATTGAGTAGAGCAGCCTTTG
Rorc +7186	TGTTCCATCGCAATGAATATG	CCCAGGCTGTCTTCCTTAGA
Cyclo	TGCCATCGCCAAGGAGTAG	TGCACAGACGGTCACTCAAA
