# Supplementary material for: The Fos-Related Antigen 1–JUNB/Activator Protein 1 Transcription Complex, a Downstream Target of Signal Transducer and Activator of Transcription 3, Induces T Helper 17 Differentiation and Promotes Experimental Autoimmune Arthritis
Source: Front Immunol. 2017 Dec 18;8:1793. doi: 10.3389/fimmu.2017.01793 (PMC5741610; doi:10.3389/fimmu.2017.01793)
Supplement: Supplementary file 4 [file Data_Sheet_1.PDF]

AP-1 Subtype Switching to FRA1-JUNB, a Downstream Target of STAT3, Induces Th17 Cell Differentiation and Experimental Autoimmune Arthritis

Young-Mee Moon[1]*, Seon-Yeong Lee[1]*, Seung-Ki Kwok[2]*, Seung Hoon Lee[1]*, Deokhoon Kim[3], Woo Kyung Kim[3], Yang-Mi Her[1], Hea-Jin Son[1], Eun-Kyung Kim[1], Jun-Geol Ryu[1], Hyeonbeom Seo[1], Jeong-Eun Kwon[1], Sue-Yun Hwang[4], Jeehee Youn[5] , Rho H. Seong[6], Sung-Hwan Park[2], Ho-Youn Kim[2], Sung-Min Ahn[3†], Mi-La Cho[1†]

[1] Laboratory of Immune Network, The Rheumatism Research Center, College of Medicine, The Catholic University of Korea, Seoul, 137-701, South Korea

[2] Center for Rheumatic Disease, Division of Rheumatology, Department of Internal Medicine, Seoul St. Mary's Hospital, College of Medicine, The Catholic University of Korea, Seoul, 137-701, South Korea.

[3] Asan Institute for Life Sciences, Asan Medical Center, University of Ulsan College of medicine, Seoul, Korea..

[4] Department of chemical engineering, Hankyong National University, Anseong, 456-749, South Korea.

[5] Department of Biomedical Sciences, College of Medicine, Hanyang University, Seoul 133-791, Korea

[6] Department of Biological Sciences, Institute of Molecular Biology and Genetics, Research Center for Functional Cellulomics, Seoul National University, Seoul 151-742, South Korea

Authorship note:

***** These authors contributed equally to this work

[†] These authors contributed equally to this work

Correspondence

†Mi-La Cho, PhD.

Rheumatism Research Center, College of Medicine, The Catholic University of Korea, 505 Banpo-dong, Seocho-gu, Seoul 137-701, Korea (South)

Phone: 82-2-2258-7467 , Fax: 82-2-599-4287

E-mail: iammila@catholic.ac.kr.

† Sung-Min Ahn, M.D, PhD

Department of Genome Medicine and Science, College of Medicine, Gachon University, Seongnam, Republic of Korea

Phone: 82-2-3010-3221 Fax: 82-2-2258-7473

E-mail: smahn@gachon.ac.kr

Supplementary Materials and Methods

Cell preparation and in vitro differentiation of Th17 cells. Splenocytes were obtained by pressing splenic tissue through a 40-μm mesh. Any red blood cells were then lysed using ACK (Ammonium-Chloride-Potassium) Lysing Buffer. To purify the $CD4^+$ T cells, the cell suspension was incubated with CD4-coated magnetic beads (Miltenyi Biotec, Bergisch Gladbach, Germany), and the bound cells were isolated using magnetic-activated cell sorting separation (MACS) columns (Miltenyi Biotec). Naïve T cells were isolated by sorting $CD4^+CD62L^+$ cells from the splenic suspension using a fluorescence-activated cell sorting (FACS) Aria III cell sorter (BD Biosciences, CA, USA). These cells were then used for Th cell differentiation and RNA-Seq studies. To generate Th17 cells, naïve $CD4^+$ T cells were stimulated by plate-bound anti-CD3 (0.5 μg/ml) and anti-CD28 (1 μg/ml) antibodies in the presence of anti-IFN-γ (2 μg/ml), anti-IL-4 (2 μg/ml), and anti-IL-2 (2 μg/ml) antibodies, and 20 ng/ml IL-6 (R&D Systems, Minneapolis, MN) and 2 ng/ml tumor growth factor (TGF)-β1 (PeproTech, Rocky Hill, NJ, USA) for 3 days. LBRM cells were transfected with the over-expression vectors to generate cell lines exhibiting over-expression using Magnetofection (Chemicell, Berlin, Germany). After 24 h, the cells were screened for neomycin resistance by culturing for 3 weeks in a medium containing neomycin (10 μg/ml). The neomycin-containing medium was changed every 3 days. Human peripheral blood (PB) was obtained from healthy donors or patients with RA. Synovial fluid (SF) was obtained from patients with RA. The diagnosis of RA was based on the 1987 revised criteria of the American College of Rheumatology [48]. PBMCs and SFMCs were isolated by density centrifugation using Ficoll-Hypaque (Pharmacia LKB, Uppsala, Sweden).

Intracellular staining and immunofluorescence. Cells were stimulated for 4 hr with phorbol 12-myristate 13-acetate (PMA) and ionomycin (both from Sigma, St. Louis, MO, USA) in the presence of GolgiStop (BD Pharmingen, CA, USA). The cells were then analyzed using a FACS Calibur (BD Pharmingen). The events were collected and analyzed with FlowJo software (TreeStar, Ashland, OR, USA). Differentiated Th17 cells were centrifuged onto slides using a Cytospin III (Thermo Scientific, MA, USA) and examined under a Zeiss microscope (LSM 510 Meta; Carl Zeiss, Oberkochen, Germany). Stained sections of frozen spleen tissue were analyzed using a confocal microscopy system (LSM 510 Meta; Carl Zeiss, Thornwood, NY, USA).

Retroviral transduction. Human *FOSL1* (GenBank accession NM_005438.3) synthesized by GenScript Corporation, with codon optimization for expression in mammalian cells was cloned into the retroviral pMX-IG vector. The retroviral vectors were transfected into Phoenix cells using X-tremeGENE DNA transfection reagents (Roche Applied Science, Penzberg, Germany). $CD4^+$ T cells were activated with plate-bound anti-CD3 (0.5 μg/ml) and anti-CD28 (1 μg/ml). The following day, the T cells were infected with retroviruses harboring FOSL1 or a control empty vector. After 12 hr, the cells were differentiated under Th17-polarizing conditions for 3 days and then re-stimulated with PMA and ionomycin in the presence of GolgiStop for 4 hr. They were then stained for surface expression of CD4 and intracellular expression of IL-17.

Dual-luciferase assay. A construct containing the *Il17a* promoter and the CNS5 (also called CNS2) firefly luciferase reporter (plasmid #20128) was used (Addgene, Cambridge, MA, USA). EL4 cells were transfected with the luciferase reporter construct or with a control Renilla luciferase reporter plasmid using Magnetofection (Chemicell) according to the

manufacturer instructions. After 24 h, the cells were cultured with phorbol myristate acetate (PMA) (25 ng/ml) and ionomycin (250 ng/ml) for a further 4 h. The dual-luciferase reporter system (Promega, WI, USA) was then used to examine the firefly and Renilla luciferase activity. Renilla luciferase was used to normalize both the transfection efficiency and the luciferase activity.

Measurement of IgG concentrations and collagen-specific IgG titers. The serum concentration of IgG, IgG1, and IgG2a was measured by ELISA using commercially available kits (Bethyl Laboratories, Montgomery, TX, USA). To measure collagen-specific IgG, IgG1, and IgG2a levels, flat-bottomed plates were coated with bovine CII and incubated overnight at 4℃. Serially diluted serum samples were then loaded into the wells and incubated at room temperature for 1 hr. The wells were then washed with washing buffer (PBS containing 50 mM Tris, 0.14 M NaCl and 0.05% Tween 20), followed by horseradish peroxidase (HRP)-conjugated goat anti-mouse IgG, IgG1, or IgG2a antibodies (Bethyl Laboratories). HRP activity was measured using tetramethyl benzidine (eBioscience, San Diego, CA, USA). The absorbance was measured at 450 nm.

Mixed lymphocyte reaction. Spleen cells were isolated from CIA-induced mice and cultured with anti-CD3 (0.5 μg/ml), CII (100 μg/ml), or MOG peptide (100 μg/ml). Eighteen hours before the end of the culture period, 0.5 μCi of [^{3}H] thymidine (New England Nuclear, Boston, MA, USA) was added to each well. The cells were harvested onto glass fiber filters and counted in a Matrix-96 direct ionization counter (Packard Instrument Co., Downers Grove, IL, USA). Data are presented as the mean cpm of triplicate cultures.

Immunohistochemistry. Joint tissues were excised from CIA and *Fra1/Junb* Tg mice .

Tissues were fixed in 4% formalin, decalcified in EDTA, and then embedded in paraffin wax. The sections were then stained with hematoxylin and eosin (H&E), Safranin O, or TRAP stain. Immunostaining was performed as described previously (46) and signals were detected using the Vectastain ABC kit (Vector Laboratories, Burlingame, CA, USA). Tissues from the joints of CIA mice or from the small intestine of GVHD mice were incubated with primary anti-IL-17, anti-IL-1β, anti-TNFα, anti-IL-6, anti-VEGF, or anti-RANKL mAb overnight at 4℃. The primary antibodies were detected with an anti-biotin rabbit or anti-biotin goat secondary antibody followed by incubation with a streptavidin-peroxidase complex for 1 hr. Color development was assessed using DAB as the chromogen (Dako, Carpinteria, CA, USA).

Histological evaluation. Inflammation was scored as follows: 0 = no inflammation; 1 = slight thickening of the lining layer or evidence of some cells infiltrating the sub-lining layer; 2 = slight thickening of the lining layer with mild cellular infiltration in the sub-lining layer; 3 = thickening of the lining layer, infiltration of cells into the sub-lining layer, and the presence of cells in the synovial space; and 4 = marked infiltration of the synovium by inflammatory cells. The following scoring system was used to assess the level of cartilage damage: 0 = no destruction; 1 = minimal erosion with damage limited to isolated areas; 2 = slight to moderate erosion in limited areas; 3 = extensive erosion; and 4 = general destruction.

Induction of CIA. CIA was induced in DBA/1J or C57BL/6 mice by intradermally injecting 100 μg of bovine CII in complete Freund's adjuvant (CFA; 50 µl) at the base of the tail. The following day, three independent observers assessed the arthritis severity. These observations were repeated three times per week for 10 weeks. The arthritis severity was recorded (on a scale of 0–4) using the mean arthritis index. The final values represent the average score from

all four legs.

Gene delivery to CIA mice. Seven days after immunization with collagen, CIA-induced mice were injected intravenously with 2 ml of saline solution containing 100 μg of the sh*Fra1/*sh*Junb* expression vectors (Santa cruz, TX, USA) over a 10-second period for hydrodynamic-based procedures. After 8 days, the same mice were injected (intramuscularly in the thigh) with 100 μg of the sh*Fra1/*sh*Junb* vectors with electrical stimulation (electroporation).

RNA-Seq and ChIP-Seq–analysis. Total RNA was extracted from naïve T cells and Th17 cells using the TRI Reagent (Molecular Research Center, OH, USA), and sequencing libraries were constructed according to the manufacturer instructions (Illumina, CA, USA). Single-end sequences (76 bp) were generated using the Illumina GAIIx. Each FastQ file was then mapped to the mouse reference genome (mm9) using Bowtie with default parameters [49]. Differentially expressed genes were identified using Cufflinks. Pathway analysis was performed using DAVID [50] and the NCI pathway interaction database [51]. STAT3 ChIP-Seq data for CD4+ T cells were downloaded from the NCBI Sequence Read Archive (SRA) (SRP002451). Each SRA file was converted into the FastQ file format, and each FastQ file was mapped onto the mouse reference genome (mm9) using Bowtie. STAT3 binding peaks were identified using a model-based analysis of ChIP-Seq [52]. We used all of the STAT3 binding sites with an annotated UCSC refGene transcriptional start site within 5 kb. A list was compiled of genes that were differentially expressed in Th17 and naïve T cells using the ChIP-Seq analysis. Both RNA-Seq and ChIP-Seq results were visually validated using the Integrative Genomics Viewer with a normalized binary alignment map format (BAM) [53].

ChIP and qRT-PCR assays. Th17 cells were cross-linked with 1% formaldehyde for 15 min at room temperature. The reaction was stopped with 125 mM glycine for 5 min. After washing three times with PBS, the cells were resuspended in a lysis buffer containing 1% sodium dodecyl sulfate (SDS) and 10 mM ethylenediamine tetraacetate (EDTA) and sonicated using a Bioruptor (Diagenode, NY, USA). The cell lysate (1×10^7 cells) was sonicated for 40 min using a pulse (30 s on followed by 30 s off). ChIP was performed overnight at 4℃, using 30 µl of Dynabeads protein A (Invitrogen) and 3 µg of anti-FRA1, anti-JUNB (Santa Cruz Biotechnology, CA, USA), anti-STAT3, or anti-IgG (Cell Signaling Technology, MA, USA). The DNA fragments were purified using a QIAquick PCR Purification Kit (Qiagen, Shanghai, China) and analyzed using quantitative qRT-PCR (Applied Biosystems, CA, USA). The primer sequences for the enriched DNA are listed in Table S3. The putative promoter region and binding regions of each gene were predicted using the Transcriptional Regulatory Element Database (TRED) and EpiTect ChIP Search Portal of SABiosciences (Valencia, CA, USA). For qRT-PCR, total RNA was extracted using TRI Reagent according to the manufacturer's instructions. RNA concentrations were measured using a NanoDrop ND-1000 (Thermo Fisher Scientific, MA, USA). Total RNA (2 µg) was reverse-transcribed using the Transcriptor First Strand cDNA Synthesis Kit (Roche Applied Science). All primers were designed using Primer-BLAST for target specific primers. mRNA expression was estimated using qRT-PCR using StepOnePlus (Applied Biosystems).

Immunoblotting and EMSA. Cells were lysed in a radioimmunoprecipitation assay (RIPA) buffer containing a protease inhibitor cocktail and a phosphatase inhibitor (Thermo Scientific Pierce, IL, USA). Proteins were separated using SDS-polyacrylamide gel electrophoresis (PAGE), and transferred to a Hybond enhanced chemiluminescence (ECL) membrane (GE Healthcare, PA, USA) for western blot analysis using the SNAP i.d.® Protein Detection

System (Millipore, Billerica, MA, USA). Whole cellular extracts were prepared from cells cultured under Th17-polarizing conditions for 3 days. EMSA analysis was performed using a $^{[\gamma\text{-}32P]}$dATP-labeled AP-1 probe (nucleotide sequence: 5'-AGCCCACTGACTCATGAGC-3' from the *Il17a* promoter; Amersham Pharmacia Biotech, Uppsala, Sweden) and the T4 polynucleotide kinase (Takara, Shiga, Japan) according to the manufacturer instructions. Unincorporated isotopes were removed using NucTrap purification columns (Stratagene, La Jolla, CA, USA). For each assay, the probes were incubated with 40 μg of cellular extract. A competitor-supershift assay was performed incorporating anti-FRA1 or anti-JUNB antibodies and an excess of unlabeled AP-1 probe to examine the binding of FRA1 or JUNB to the AP-1 probe. Briefly, cellular extracts were incubated with anti-FRA1 and/or anti-JUNB for 15 min on ice before adding the AP-1 probe. The samples were then electrophoresed on non-denaturing 5% polyacrylamide gels in 0.5 × Tris-Borate-EDTA buffer at 100V. The gels were then vacuum dried and exposed to Kodak X-OMAT film plus intensifying screens for 12 to 24 h at −70°C.

Statistical analysis. Data were analyzed using the GraphPad Prism 4.0 software and are presented as the means ± standard error of the mean (SEM) of more than two independent experiments. Data were compared using an unpaired t-test, one or two-way ANOVA with Bonferroni's post-test. A P value of < 0.05 was considered to indicate significance.
