# Supplementary figures and images for: The Fos-Related Antigen 1–JUNB/Activator Protein 1 Transcription Complex, a Downstream Target of Signal Transducer and Activator of Transcription 3, Induces T Helper 17 Differentiation and Promotes Experimental Autoimmune Arthritis
Source: Front Immunol. 2017 Dec 18;8:1793. doi: 10.3389/fimmu.2017.01793 (PMC5741610; doi:10.3389/fimmu.2017.01793)

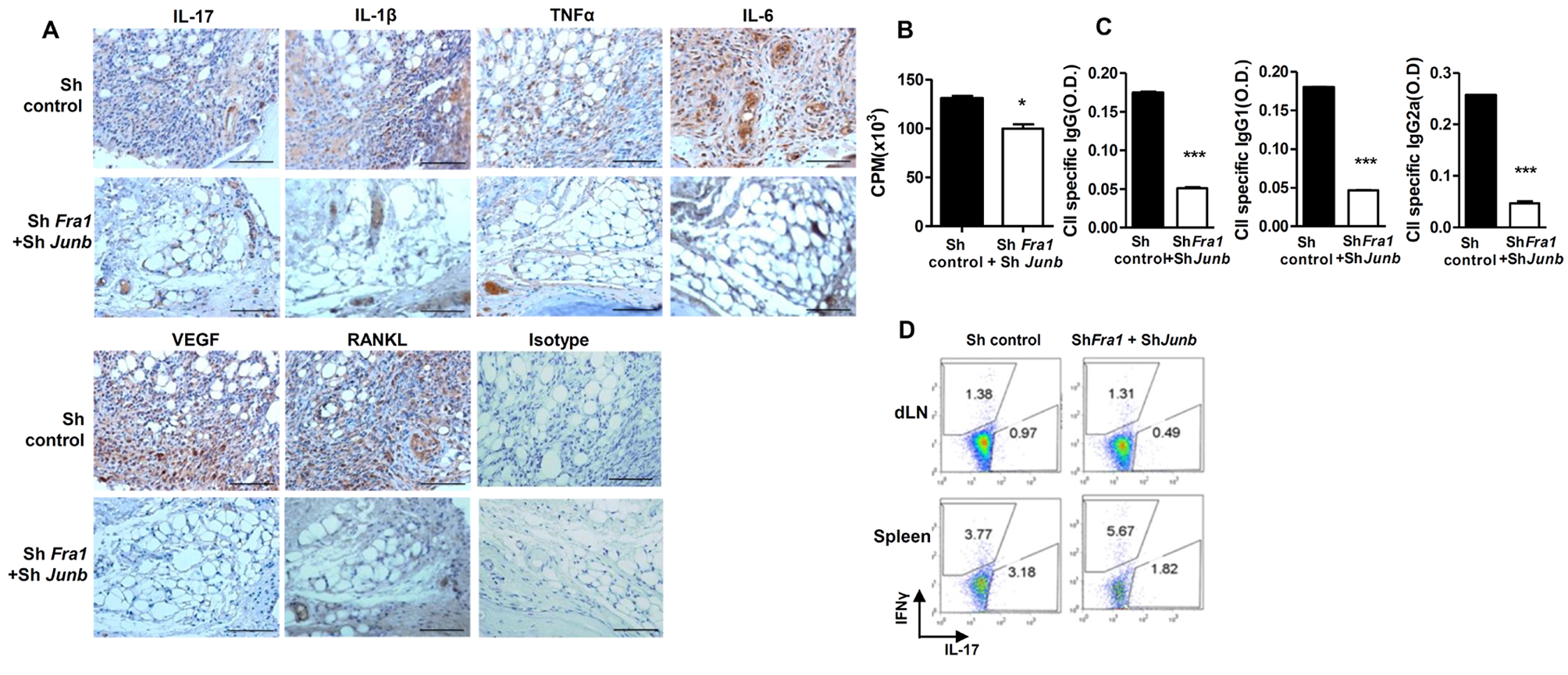

Supplement: Figure S1 — Knockdown of FRA1/JUNB Decreases Th17 Cell Differentiation and Attenuates Rheumatoid Inflammation in Mice. [file Image_1.tif]

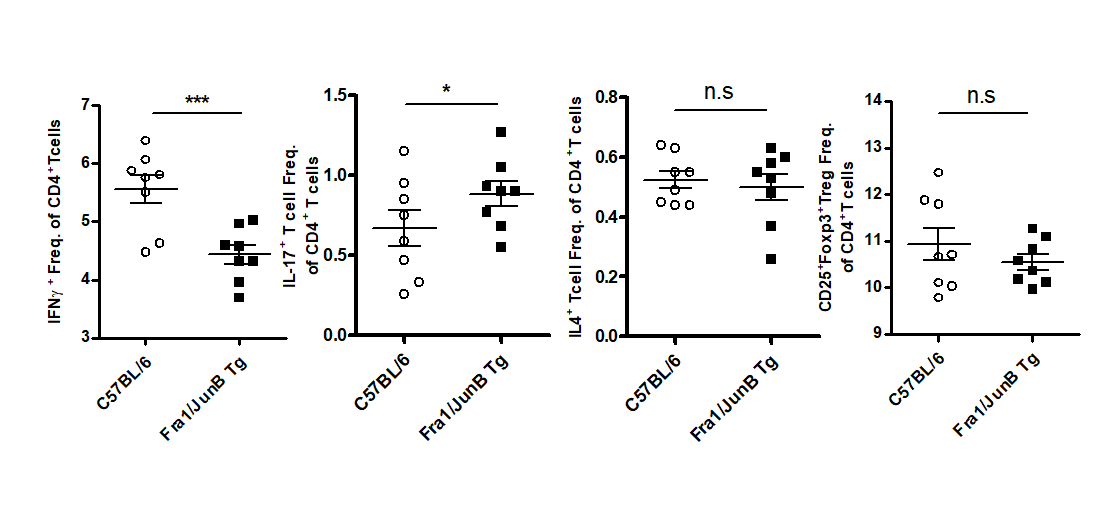

Supplement: Figure S2 — T cell proportion of CD4+Tcells in Fra1/Junb Tg mice. [file Image_2.tif]

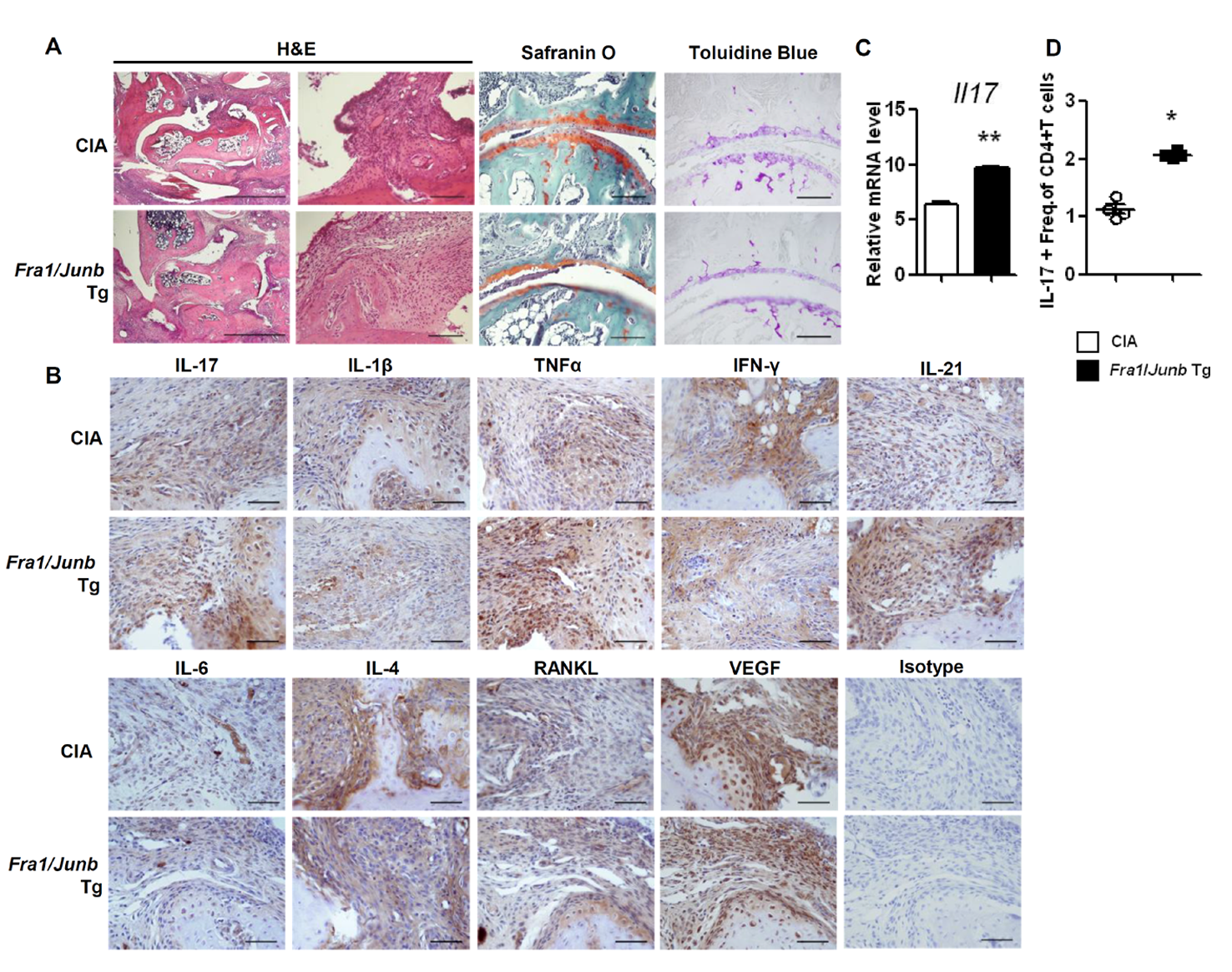

Supplement: Figure S3 — Overexpression of FRA1/JUNB Causes Th17 Cell Differentiation and Increases CIA Disease Severity. [file Image_3.tif]
